# Are Metal Concentrations in Lesser Flamingo Eggs and Eggshells Good Indicators of Environmental Pollution?

**DOI:** 10.1007/s00244-024-01102-9

**Published:** 2024-11-27

**Authors:** Nicole van Gessellen, Hindrik Bouwman

**Affiliations:** Research Unit: Environmental Sciences and Management, North–West University, Potchefstroom, South Africa

## Abstract

Metallic elements in excess may cause adverse biological effects. Flamingos, with a lifespan of up to 50 years, are therefore likely to accumulate metals from the highly saline waters where they feed and breed. The concentrations of accumulated metals would be reflected in organs, feathers, and eggs. There are no data available on metals in flamingo egg contents. Concentrations of 24 elements in egg content and eggshells from Lesser Flamingo *Pheoniconaias minor* breeding at Kamfers Dam South Africa suggest metal pollution, but with considerable variation between eggs, reflecting their nonbreeding, nomadic movements. Strontium in eggshells exceeded toxic reference values. Copper in egg contents suggests reproductive stress. Lower than-expected metal concentrations (especially mercury) in egg contents we attributed to several excretory pathways prior to oogenesis and embryogenesis, thereby protecting the embryo. Molar concentrations of selenium and mercury were not correlated. However, the mean molar ratio of 8.2 suggests a highly protective effect afforded by selenium. Relative compositional differences show that eggshells are not a proxy for egg contents. We highlight previously unrecognised routes of post-hatching pollutant uptake via consumption of eggshells and parental crop milk. The post-hatching development of flamingo chicks may therefore be more susceptible to pollutant disruption compared with embryonic development. We conclude that *P. minor* eggs are not good indicators of environmental metal pollution, nor does it reflect post-hatching risks. This first report on metal concentrations in egg contents of any flamingo species shows that a more nuanced approach is needed to protect Phoenicopteridae from pollution.

Flamingos (family Phoenicopteridae) are of the oldest bird taxa, with six extant species (Colyn et al. 2024). They have a long cultural history with humans, depicted in cave paintings from about 5000 BCE (Kear and Duplaix-Hall 1975; Krienitz 2018), hieroglyphs in Egypt, and rock paintings and engravings in southern Africa (Krienitz 2018). The Lesser Flamingo *Phoeniconaias minor* is one of two flamingo species in Africa (Kear and Duplaix-Hall 1975; McCulloch et al. 2003; Simmons 1996) and is important conservation flagship birds (Krienitz 2018).

Lesser Flamingos inhabit shallow, eutrophic, open, and saline water bodies such as salt pans, coastal estuaries, and lagoons across sub-Saharan Africa and India (BirdLife International 2023; BirdLife South Africa 2018a; Krienitz and Kotut 2010). They are adapted to harsh environments and have a specialised diet of cyanobacteria, benthic diatoms, and small aquatic invertebrates (Childress et al. 2008; Hill 2016; Hockey et al. 2005). In southern Africa, Lesser Flamingos breed at three sites: Sua Pan in Botswana, Etosha Pan in Namibia, and Kamfers Dam, Kimberley, South Africa (Krienitz 2018). Kamfers Dam probably supports the largest population of *P. minor* in southern Africa, sometimes with more than 80 000 individuals (Anderson and Anderson 2010). It is therefore recognised as an Important Bird and Biodiversity Area (BirdLife South Africa 2018b).

Flamingos are among the few birds that feed and breed in highly saline water bodies and would be useful biological indicators of this ecosystem’s general health (Krienitz and Kotut 2010). As filter feeders, they filter large amounts of water and sediment through their mouths using their tongues as a pump, bringing them into chronic contact with high amounts of metal salts (Krienitz 2018; Sherry 2016; Tenai et al. 2016). In addition, Lesser Flamingos also live up to 50 years (Krienitz 2018). Their longevity and size, therefore, should make them good indicators of pollutants accumulated over their lifetime (Barbieri et al. 2010; Ebert et al. 2020).

Unfortunately, the southern African population of Lesser Flamingos are declining (Colyn et al. 2024; Hill 2016; Simmons 1996; Simmons 2000) and have been placed on the IUCN Red List as Near Threatened (BirdLife International 2023). Significant threats to Lesser Flamingo at Kamfers Dam include degradation and loss of its habitat due to altered hydrology and worsening water quality (BirdLife SA 2018a; Childress et al. 2008; Hill et al. 2013; Roos 2008b). High concentrations of DDTs, PCBs, and other organochlorines in the tissues of the Greater Flamingos *P. ruber* from Spain (Guttiérez et al. 1997) and *P. minor* from Kenya (Kairu 1994) show that pollutants enter flamingos from water and food from salty waters. Elsewhere in Africa, there have been reports of mass die-offs of *P. minor* associated with malnutrition (Krienitz and Kotut 2010), cyanobacterial toxins (Ballot et al. 2005; Krienitz and Kotut 2010; Nonga et al. 2011), ‘heavy’ metals (Ndetei and Muhandiki 2005; Nelson et al. 1998), and pesticide usage making them more vulnerable to diseases or other stressors (Krienitz et al. 2005; Straubinger-Gansberger et al. 2014; Zimmermann et al. 2011).

Metals occur naturally in the environment (Newman 2015; Nordberg et al. 2014). However, metals are also well-known environmental contaminants. They enter the environment through anthropogenic activities such as industrial processes, urban and suburban runoff, agriculture (Briffa et al. 2020), or combinations thereof. Many metals are essential and are incorporated into biota for various physiological functions (Gillingham et al. 2021). Examples are iron (Fe), zinc (Zn), copper (Cu), and manganese (Mn) (Adams et al. 2020; Valiente et al. 2022). Some elements (e.g. arsenic (As), nickel (Ni), and vanadium (V)) are essential in some organisms such as bacteria, plants, and animals, but most of their physiological functions are not known (Newman 2015; Nordberg et al. 2014). Metals such as cadmium (Cd), lead (Pb), and mercury (Hg) are non-essential. However, any metal can become toxic at concentrations elevated beyond an organism’s physiological and homeostatic control (Aloupi et al. 2017; Gillingham et al. 2021; Newman 2015). Non-essential elements such as Pb, Cd, As, and Hg can be taken up by some organisms faster than they can be eliminated and, as a result, can bioaccumulate (Newman 2015). Koivula and Eeva (2010) further list harmful effects of metals on birds (Table 1).

**Table 1:** Concentrations (mg/kg) of dry mass and metals in *P. minor* eggs from Kamfers Dam in 2008

		B	Mg	Al	Ti	V	Cr	Mn	Fe	Co	Ni	Cu	Zn	As	Se	Rb	Sr	Mo	Pd	Ag	Cd	Sb	Ba	Hg	**Pb**
Shell	*n* > LOQ	11	11	11	11	5	10	11	11	11	11	11	11	0	0	11	11	0	11	4	0	0	11	11	11
	Min	35	1400	33	5.7	0.14	0.12	13	570	1.0	0.9	2.9	6.3	ND	ND	0.2	650	ND	0.68	0.002	ND	ND	8.1	ND	0.13
	Max	43	2200	440	18	1.5	4.6	160	910	1.9	2.7	16	44	ND	ND	0.8	990	ND	1.1	0.074	ND	ND	24	ND	1.2
	Median	40	1800	60	10	0.25	1.5	21	700	1.2	1.1	6.2	9.6	ND	ND	0.3	880	ND	1.0	0.025	ND	ND	13	ND	0.45
	Mean	39	1900	99	10	0.47	1.7	33	690	1.2	1.3	6.6	16	ND	ND	0.4	840	ND	0.94	0.032	ND	ND	15	ND	0.54
	SD	2.7	270	120	4.1	0.6	1.3	43	97	0.2	0.5	3.4	13	ND	ND	0.2	120	ND	0.16	0.031	ND	ND	5.4	ND	0.31
	%CV	6.8	14	120	40	130	76	130	14	19	39	52	84	ND	ND	48	15	ND	17	96	ND	ND	37	ND	58
Content	*n* > LOQ	11	11	11	11	11	11	11	11	11	11	11	11	8	11	11	11	11	9	11	6	11	11	11	11
	Min	4.3	160	1.5	4.7	0.00032	0.77	0.46	26	0.053	0.11	2.3	29	0.018	0.23	3.2	3.7	0.037	0.00083	0.032	0.00042	0.00081	0.34	0.11	0.07
	Max	7.5	1300	13	9.0	0.16	2.8	3.8	130	0.093	0.79	4.5	95	0.60	0.83	6.0	24	0.24	0.016	0.28	0.023	0.0087	0.69	0.24	0.34
	Median	4.8	500	2.0	7.4	0.061	1.4	1.7	75	0.076	0.25	3.4	56	0.15	0.46	5.4	12	0.092	0.0046	0.08	0.0031	0.0025	0.52	0.17	0.18
	Mean	5.0	570	3.0	7.0	0.062	1.7	1.9	78	0.077	0.31	3.3	53	0.22	0.50	4.9	14	0.11	0.0064	0.13	0.0057	0.0033	0.51	0.17	0.18
	SD	0.91	320	3.2	1.3	0.046	0.8	1.0	32	0.013	0.21	0.71	19	0.20	0.18	1.0	6.5	0.055	0.006	0.087	0.0085	0.0024	0.11	0.047	0.092
	%CV	18	56	110	18	75	44	55	42	17	66	22	36	90	36	20	48	52	87	70	150	71	22	28	51

ND = below limits of quantification

< LOQ (below limit of quantification)

Bird species have homeostatic mechanisms to protect them from metals (Scherer et al. 2015). Metals can be excreted via urine, faeces, supraorbital and uropygial glands, sequestered in feathers, eliminated via eggs, and combinations thereof (Burger and Gochfeld 2001; McWilliams et al. 2016). Metal concentrations in eggs are derived from prior and recent uptake, and from mobilisation from other tissues (Burger et al. 1999; Dauwe et al. 2005). Concentrations in bird eggs, therefore, may serve as indicators of female exposure from prior and nearby sources, potential reproductive and hatchling impacts, and function as an early warning for potential ecosystem effects when concentrations are elevated (Burger and Elbin 2015).

Pollutants in bird egg content should reflect those in the maternal bloodstream and organs before and during oogenesis and embryogenesis (Hargreaves et al. 2010). Eggshells may provide additional information since adults transfer some contaminants and elements during egg development although species-specific caveats apply (Mora 2003; Rocha et al. 2021). To the best of our knowledge, there are no publications on metals in flamingo egg contents and only few on eggshells (Table 2).

**Table 2:** Concentrations (mg/kg dm) of metallic elements in studies of flamingos and other birds from the same region as our collection

Species	Location	Matrix	Cd	Hg	Cu	Pb	Zn	Se	Cr	Mn	As	Ba	Ni	Fe	V	Ti	Pd	Source
*Phoeniconaias ruber*	Italy	Liver	1.61	6.77	NM	361.29	NM	NM	NM	NM	NM	NM	NM	NM	NM	NM	NM	Ancora et al. (2008)
		Kidney	9.8	2.08	NM	238.71	NM	NM	NM	NM	NM	NM	NM	NM	NM	NM	NM	
		Bone	0.03	0.17	NM	43.32	NM	NM	NM	NM	NM	NM	NM	NM	NM	NM	NM	
		Muscle	0.18	1.06	NM	4.1	NM	NM	NM	NM	NM	NM	NM	NM	NM	NM	NM	
		Feathers	0.07	2.36	NM	1.66	NM	NM	NM	NM	NM	NM	NM	NM	NM	NM	NM	
*P. minor*	Namibia	Feathers	0.038	0.077	NM	0.386	NM	0.841	0.682	2.31	0.976	NM	NM	NM	NM	NM	NM	Burger and Gochfeld (2001)
*P. minor*	South Africa	Blood*	1.53	NM	NM	2.48	NM	NM	NM	NM	NM	NM	NM	NM	NM	NM	NM	Hill (2016)
		Feathers*	0.26	0.42	64.32	13.75	140.66	0.46	0.82	1.94	NM	22.37	1.58	NM	NM	NM	NM	
*P. roseus*	Spain	Muscle	0.04	0.088	24	0.025	56.2	NM	NM	NM	NM	NM	NM	NM	NM	NM	NM	Valiente et al. (2022)
		Liver	0.304	0.534	34.6	0.16	382	NM	NM	NM	NM	NM	NM	NM	NM	NM	NM	
		Fat	0.007	< LOQ	1.87	0.075	14	NM	NM	NM	NM	NM	NM	NM	NM	NM	NM	
*P. andinus*	Bolivia	Eggshells*	< LOQ	< LOQ	7.2	< LOQ	6.2	NM	NM	NM	7.8	NM	NM	23	NM	NM	NM	Rocha et al. (2021)
*P. jamesi*			< LOQ	< LOQ	5.8	< LOQ	5.05	NM	NM	NM	8.9	NM	NM	23	NM	NM	NM	
*P. ruber*	Spain	Liver	0.55	NM	56	149	403	NM	1.1	13	NM	NM	NM	NM	NM	NM	NM	Mateo and Guitart 2003
*P. ruber*	France	Feathers	0.06	NM	4.31	3.59	90.5	0.85	NM	NM	NM	NM	NM	NM	NM	NM	NM	Amiard-Triquet et al.(1991)
*Ardea cinerea*	Baberspan (South Africa)	Egg contents	0.15	7	7	1.6	65	10	5	3.2	2.3	NM	2.1	230	2.4	7.5	6.5	Van der Schyff et al. (2016)
*A. cinerea*	Bloemhof Dam (South Africa)		0.072	3.6	8.3	0.56	61	6.1	6.7	2.4	2.1	NM	1.3	220	0	< LOQ	< LOQ	
*Threskiornis aethiopicus*	Bloemhof Dam (South Africa)		0.031	1.3	6.8	0.62	68	4.6	8.9	3.7	2.2	NM	1.5	220	2.7	5.3	0.74	
*T. aethiopicus*	Soweto (South Africa)		0.038	2.1	10	0.6	65	5.5	7	3.8	1.8	NM	1.3	210	2.2	5	1.1	
*Anhinga rufa*	Baberspan (South Africa)		0.026	2.4	8.5	0.37	70	6.5	7.5	3.4	1.7	NM	0.95	180	2.8	5.2	0.51	
*A. rufa*	Bloemhof Dam (South Africa)		0.026	2.5	9.2	0.51	61	6.7	8.5	3.2	2.6	NM	1.5	200	< LOQ	< LOQ	< LOQ	
*Microcarbo africanus*	Potchefstroom (South Africa)		< LOQ	3.2	5.4	0.41	62	6.2	8.5	4.9	2	NM	1.1	200	2.2	5.5	0.85	
*M. africanus*	Bloemhof Dam (South Africa)		0.022	2.2	8.9	0.52	73	6.3	7.8	2.9	2.1	NM	1.5	220	2.9	5.7	0.79	
*Egretta alba*	Bloemhof Dam (South Africa)		0.038	9.5	7.5	0.65	75	5	6	2.6	1.9	NM	1.3	220	2.2	6.1	1.6	
*Egretta garzetta*	Bloemhof Dam (South Africa)		0.026	1.5	6	0.38	75	4.4	8	3.3	2.1	NM	1.3	230	2.7	5.2	0.52	
*Plegadis falcinellus*	Potchefstroom (South Africa)		< LOQ	1.6	5	0.48	70	7	8	5	2.2	NM	1.2	200	< LOQ	< LOQ	< LOQ	
***P. minor***	**South Africa**	Eggshells*	** < LOQ**	** < LOQ**	**6.2**	**0.45**	**9.6**	** < LOQ**	**1.7**	**21**	** < LOQ**	**13**	**1.1**	**700**	**0.25**	**10**	**1**	**Present study**
		Egg contents*	**0.0031**	**0.17**	**3.4**	**0.18**	**56**	**0.46**	**1.4**	**1.7**	**0.15**	**0.52**	**0.3**	**75**	**0.061**	**7.4**	**0.0046**	

Bold value indicates the current study’s concentrations

^*^medians

< LOQ (below limit of quantification)

NM—not measured

In this study, 24 metallic elements, as well as arsenic (As) and selenium (Se), that are metalloids, were measured in the egg contents and eggshells of 11 *P. minor* eggs from Kamfers Dam in Kimberley, Northern Cape, South Africa. The main objectives of this study were to measure concentrations, investigate relative elemental compositional differences between eggshells and egg content; determine whether metal pollution can be inferred from concentrations in the egg content from eggshells; assess whether these concentrations may have contributed to the decline in the flamingo population at Kamfers Dam; investigate if metals can affect embryo health; and more fully discuss the biology of flamingos as it may relate to pollutants. We predict that the metal concentrations in the eggs and eggshells would be high because of their long lifespans and chronic exposure to highly saline water. Since *P. minor* are widely nomadic with large ranges, but breeds at only a few sites, we also predict large variations in concentrations.

## Materials and Methods

### Site Description—Kamfers Dam

**Fig. 1:**
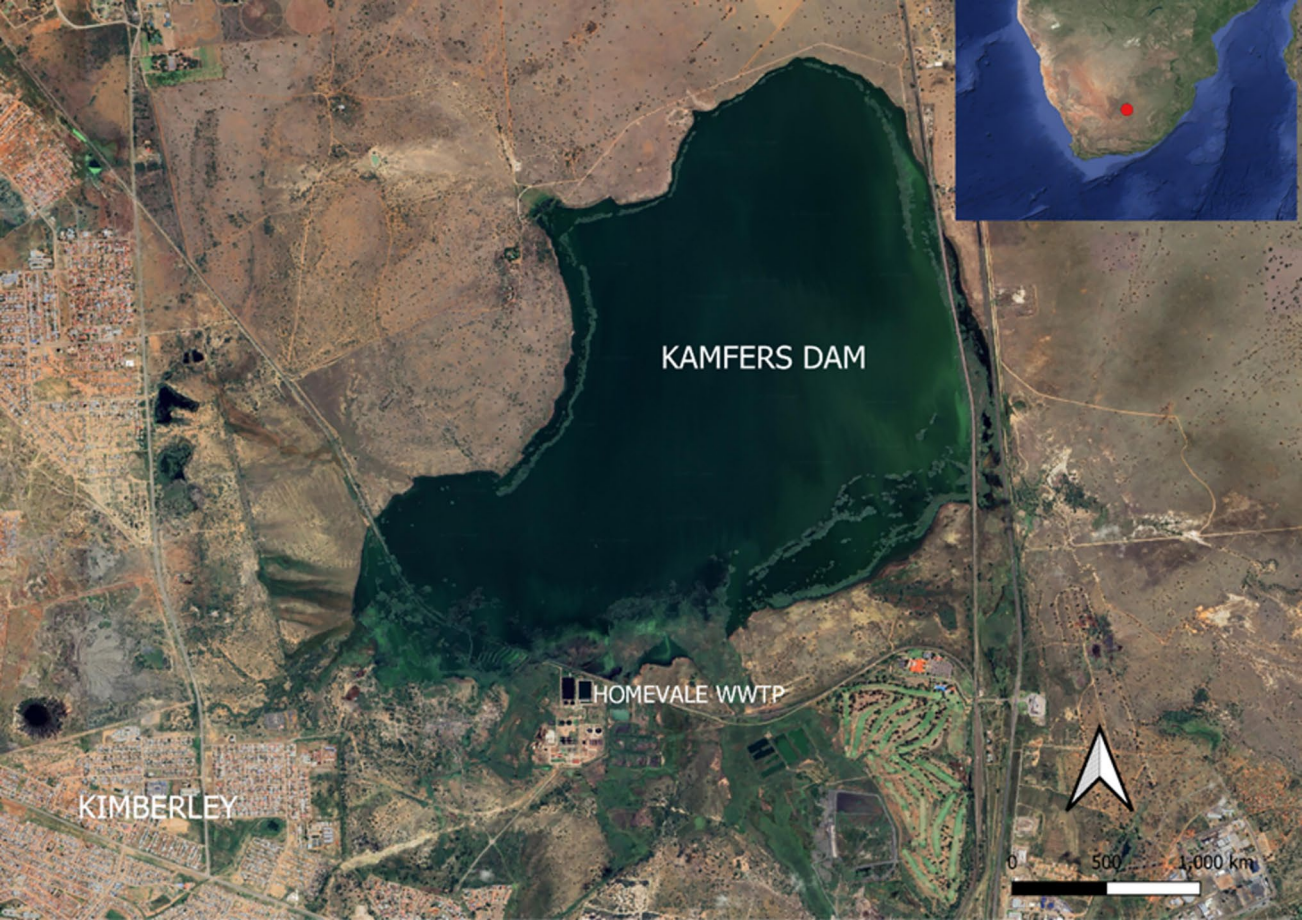
Satellite image of Kamfers Dam and its surrounds. The inset shows the location of Kimberley in South Africa as a red dot

Kamfers Dam is a 500-ha endorheic (no outflow) wetland (Fig. 1) situated 7 km north of the city of Kimberley, Northern Cape province, South Africa (Childress et al. 2008; Hill 2016). This wetland was ephemeral but is now permanently inundated as it receives most of Kimberley’s effluent from stormwater and a wastewater treatment plant (WWTP) (Anderson and Anderson 2010). This now permanent wetland is an oasis for waterbirds in the semi-arid Northern Cape (Childress et al. 2008; Ramollo 2018). The high ambient salinity and additional nutrient inputs from the WWTP cause massive algal blooms which is a major food component for flamingos (Childress et al. 2008; Hill et al. 2013; Ramollo 2018). Kamfers Dam also supports Greater Flamingos *Phoenicopterus roseus* and other water birds (BirdLife South Africa 2018b; Ramollo 2018).

**Fig. 2:**
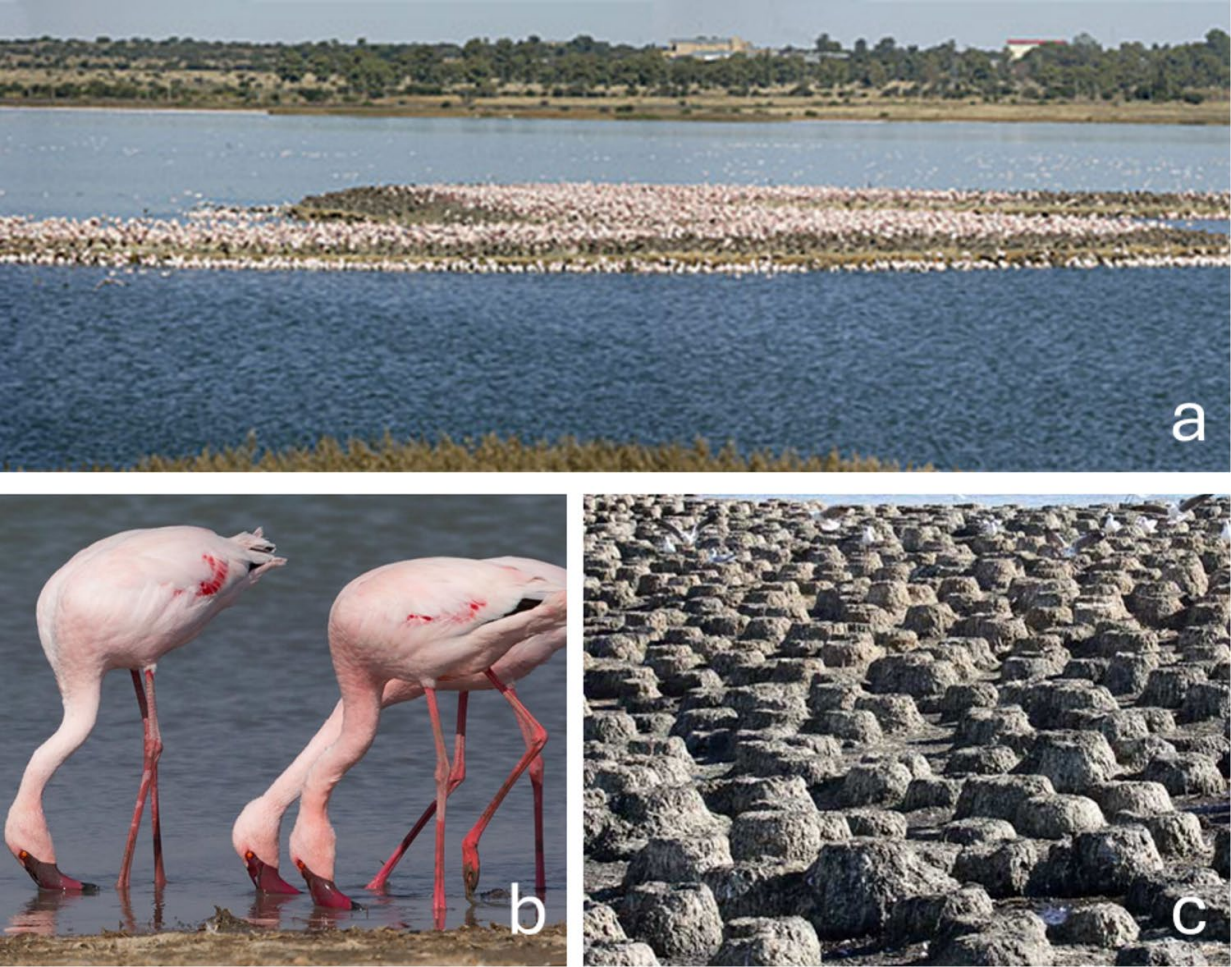
**a** Flamingo breeding island, Kamfers Dam, with Kimberley in the background, **b** flamingos filtering for food, **c** nests after breeding season. All photographs by Warick Tarboton (2022)

Most surrounding terrestrial habitat is natural, but there is also transformation due to two railway lines, roads, farm buildings, a farm dam, a waste water treatment plant (WWTP), a coal ash dump, and erosion (BirdLife South Africa 2018b). In September 2006, following months of planning and an extensive environmental impact assessment, a flamingo island (Fig. 2a) was constructed by Ekapa Mining at Kamfers Dam (Anderson and Anderson 2010). This was spectacularly successful: 9,000, 13,000, and 500 Lesser Flamingo chicks hatched in 2008, 2009, and 2010, respectively (Anderson and Anderson 2010; Childress et al. 2008).

### Species Description—*Phoeniconaias* Minor

**Fig. 3:**
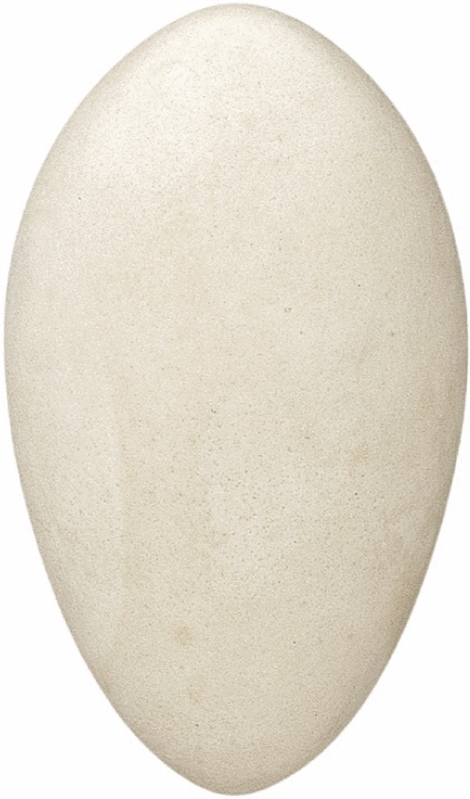
Photograph of *P. minor* egg (Tarboton 2022)

*P. minor* reach sexual maturity at 3–4 years (Fig. 2b). They breed in large flocks following seasonal rains (Simmons 1996), using soft, muddy substrate to build their nests (Fig. 2c). When not breeding, they are nomadic and roam over large areas, often in smaller flocks (Hockey et al. 2005). When they do breed at Kamfers Dam, breeding peaks in May–June (Tarboton 2014). Clutch size is one, rarely two (Tarboton 2014). The incubation period is 28 days, and the fledgling stage is approximately 70 days (Childress et al. 2008). Tarboton (2014) lists the egg dimensions (minimum-mean-maximum): 72.0–82.3–94.0 mm × 44.0–49.9–56.0 mm, and the mass at 61–97-118 g (Fig. 3).

### Sample Collection and Preparation

Egg collection and handling were approved by the Northern Cape Province Department of Environment and Nature Conservation and by the North-West University Ethics Committee (NWU-00774-24-A5). Eleven abandoned eggs were collected from Kamfers Dam in August 2008 and frozen after collection. In the laboratory, eggs were weighed and measured. Because of the frozen condition of the eggs, egg mass was the only parameter we have confidence in. The egg contents and eggshells were separated. The wet content was ultrasonically homogenised with a micro-tip probe for one minute. Care was taken not to heat the contents and to keep foaming to a minimum. The homogenised content is placed in high-density polyethylene bottles frozen. Sub-samples were freeze-dried (for 48 h at 80 °C and 0.133 Pa) to obtain a dry mass sample of ca. 0.2 g.

The eggshells were cleaned with lukewarm water after the egg content was removed. The membrane around the shell was removed by gently rubbing the inside of the shell. The shells were then air-dried for 24 h. The shells were crushed to a fine powder with a pestle and mortar.

### Chemical Analysis

Samples were analysed at the Eco-Analytica laboratory at North-West University. Twenty-nine elements were analysed. In the absence of a bird egg standard reference material, we chose a lipid- and protein-rich alternative from a saline environment (mussel tissue, ERM-CE278k). Dried sample (0.2 g) was microwave digested (Ethos UP, Magna Analytical) according to the EPA3051A method, using an Agilent ICP-MS (Agilent 7500 CE ICP-MS) fitted with a MicroMist nebuliser and standard quartz chamber. Forward power was set at 1550 W, the plasma gas flow was 15 l/min, and the nebuliser gas flow was set at 1.2 l/min.

The instrument was optimised using a solution containing Li, Y, Ce, and Tl (1 mg/L) to reduce interference and improve sensitivity across the mass range. External calibration was with ULTRASPEC-certified multi-element standard solutions (De Bruyn Spectroscopic Solutions) containing all the elements of interest. Quality control standards were used for each run. Detection limits for each element were calculated from the calibration curves with each calibration run, and quantification was done within the linear calibration range. Concentrations are expressed in mg/kg dry mass (dm) for egg contents and eggshells.

### Statistical Analysis

GraphPad Prism version 10.0.0 (www.graphpad.com) was used for most statistical analyses with log-transformed data for Spearman correlations between elemental concentrations, linear regressions between concentrations and egg mass, and a paired, two-sided t-test between log-transformed %CV (percentage coefficient of variation) values of concentrations in egg contents and eggshells. PCORD version 6.20 (MjM Software Design, www.pcord.com) was used for non-metric multidimensional scaling (NMS) between different variables, with Sørensen as the distance measure. Data were relativised per egg. Initial six dimensions were allowed, with 500 iterations from a random starting condition.

### Additional Information

We obtained technical reports on the water quality of Kamfers Dam for February, July, and November 2008, prepared for the Department of Tourism, Environment and Conservation of the Northern Cape province that we used to contextualise our findings' results.

## Results

### Concentrations

The mean egg mass was 72.1 g, with a minimum of 50.4 g, a maximum of 98.4 g, and a standard deviation (SD) of 16.8 g. Elemental recoveries were within 6% of the certified values of the standard reference mussel tissue. Most of the 29 elements analysed were quantifiable (Table 1). In the shells, Be, Se, Sb, Pt, Hg, Tl, Bi, and Th concentrations were below the limits of quantification (< LOQ). Arsenic, Cd, and U were only quantifiable in one or two samples and were not used in further calculations. In egg contents, Pt, Au, Bi, Th, and U were not quantifiable or quantifiable only once. These elements were also not used in further statistical analyses.

**Fig. 4:**
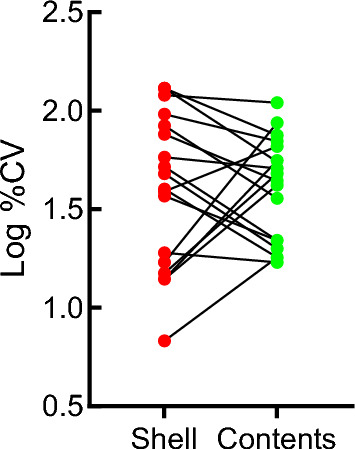
Comparison of log percentage coefficients of variation (%CV) between elemental concentrations of eggshells and egg contents (paired, two-sided, *t*-test)

The mean %CV for eggshells was 55% and 53.5% for egg contents. There was no statistical difference in mean log %CV between eggshells and egg contents when only paired elements (quantifiable in both eggshell and egg contents) were tested (*p* = 0.9407; Fig. 4). The %CV of elemental concentrations larger than 50% suggest individual differences in uptake and or accumulation (Van der Schyff et al. 2016) since *P. minor* feed nomadic over wide ranges whereafter they congregate and breed at only a few sites (Hockey et al., 2005). For both eggshells and egg contents, Al, V, Mn, Ag, and Pb exceeded 50% CV (Table 1). In shells, elements exceeding 50% were Cr, Cu, and Zn. In egg contents, these were Mg, Ni, As*, Mo*, Pd, Cd*, and Sb*. Elements marked with * were not quantifiable in eggshells.

No significant (*p* < 0.05) Spearman correlations were found between corresponding element concentrations in contents and shells. Nor were there any significant linear regressions of corresponding concentrations between shell or content regressions with egg mass. Since Hg was only quantifiable in egg contents, a Spearman correlation between Hg and Se concentrations in egg contents showed no significant association (*p* = 0.9602).

### Multivariate Analysis

**Fig. 5:**
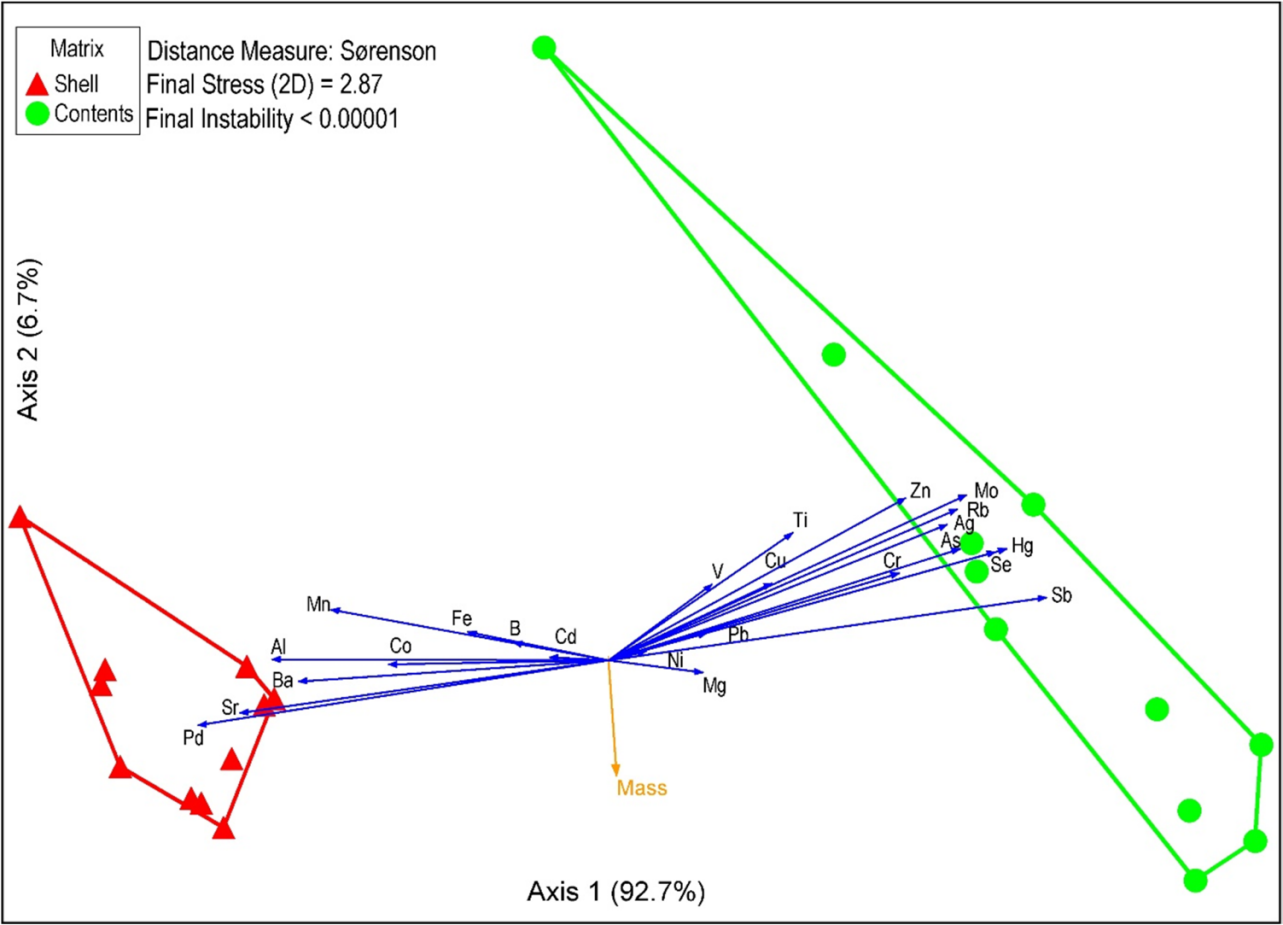
Nonmetric multidimensional scaling (NMS) of proportional compositions of metals in eggshells and egg contents using convex hulls. The egg mass vector is indicated in the biplot as mass

The non-metric multidimensional scaled ordination (Fig. 5) showed no overlap between relative compositional differences of eggshells and egg contents when looking at the respective convex hulls (encompassing the outer extents of sample ordinates). The final stress was low at 2.87 (meaning hardly any distortion of the points was needed to plot the samples and elements in two dimensions), with a final instability of < 0.00001 reached after 45 iterations. The horizontal axis explained 92.7% of the variation, with the egg mass vector in the biplot perpendicular to the elemental axis suggesting no influence of egg mass.

### Comparable Data

Table 2 presents comparable flamingo data from elsewhere, noting the absence of any egg content data. Because of the lack of flamingo egg content data, we included comparable data from other species from the same region.

## Discussion

### Concentration Comparisons Between Egg Contents and Eggshells

Although eggshells and egg contents are produced in the oviduct, they are distinct tissues (Hickman et al. 2019). Boron, Mg, Al, Mn, Fe, Co, Ni,, Sr, Pd, and Ba occur in at least one order of magnitude higher concentration in eggshells than in egg content (Table 1). Conversely, Hg, Mo, Cd, Sb, As, and Se occurred in egg contents greater than LOQ but were not quantifiable in eggshells. Peterson et al. (2017) found that the embryo’s age influences Hg concentrations in bird’s eggshells. Titanium, Cr, Cu, Ag, and Pb had similar order of magnitude concentrations in both tissues. Different elements occur in different concentrations in the two tissues, reflecting the sustenance role of the egg contents and the protective role of the eggshell.

The NMS (Fig. 5) clearly showed differences in the composition of eggshell and egg content. The eggshell had higher relative compositions of Zn, Mo, Rb, Ag, As, Se, Hg, and Sb, while the contents had higher compositions of Ba, Al, Sr, and Pd. Figure 5 shows that metal concentrations in eggshells cannot be used as proxy for egg contents. Instead, the egg contents must be used to monitor metallic element concentrations as these are physiologically relevant to the embryo’s development, with some mobilisation from the shell occurring during development (Orłowski and Hałupka 2015). The eggshell, however, cannot be ignored when considering elements relevant to the embryo. It must be included in the element budget (Orłowski et al. 2017). As will be shown later, the chicks may remobilise elements deposited in eggshells after they have hatched (Berry 1975).

On the notable absence of quantifiable Hg in eggshells: Flamingo eggs are laid out in the open exposed to harsh sunlight. Mercury is volatile despite its high molecular mass and low vapour pressure (Okouchi and Sasaki 1984). One may therefore suspect that Hg may be lost from the shells to the environment at high ambient temperatures due to volatilisation. Indeed, this may be the case for Hg in eggshells where we had no quantifiable concentrations. However, we found no corroborating indications from literature. We believe, however, that Hg in egg contents would remain stable. If Hg was lost from here, it can only be via the eggshell, where we found none, suggesting that Hg in egg contents experiences negligible loss to the environment.

### Migratory Behaviours and Elemental Variations

Lesser Flamingos may migrate hundreds of kilometres to find suitable locations to forage and breed (Colyn et al. 2024; Kaggwa et al. 2013; Krienitz 2018). In central southern Africa, they seem to be partial migrants as they often follow a regular, repeated pattern following specific routes. They frequently travel between two primary locations—Sua Pan (Botswana) and Kamfers Dam (South Africa)—for breeding. However, they also are found in smaller groups all over southern Africa far away from their breeding sites (Broekhuysen 1975; Hockey et al. 2005; Pretorius et al. 2020). The inter-individual differences in concentrations (Table 1) may reflect these migratory and semi-nomadic movements, age, and feeding and breeding histories of individuals over their lifespans, acquiring different elemental concentration profiles. Where they congregate to breed in large groups, these individual histories will manifest as concentration differences in their eggs.

More information may be obtained from the coefficients of variations (%CV; Table 1; Fig. 4). The mean %CV for elemental concentrations in eggshells (55%) and contents (53.3%) did not differ significantly (Fig. 4). The large differences in %CV between individual elements (such as Mg, V, Ni, and Zn; Table 1), however, support the assumption that large feeding ranges with assumed large differences in elemental compositions in food are reflected in widely differing %CVs in eggs where the Lesser Flamingo congregate to breed.

### Comparisons with Literature

Chromium, Fe, and Zn have been identified as potential stressors contributing to the mass mortalities observed in Lesser Flamingos in the Kenyan Rift Valley lakes (Nelson et al. 1998). Iron is notably higher in *P. minor* eggshells than eggshells from *P. andinus* and *P. jamesi* (700 mg/kg vs 23 mg/kg; Table 2). Zn concentrations were also higher in *P. minor* eggshells than those reported by Rocha et al. (2021). As in our case, Rocha et al. (2021) did not find quantifiable Hg in eggshells, while we did quantify it in egg contents (Table 2). Peterson et al. (2017) also found lower concentrations of Hg in eggshells than in egg contents for a number of other species they reviewed.

Due to the few egg-related reference points for flamingos, we compared our data with other waterbird eggs from the same catchment in South Africa (Van der Schyff et al. 2016; Table 2), all of them feeding at higher trophic levels than flamingos. All the metallic concentrations in *P. minor* egg contents were lower (up to two orders of magnitude in some cases) except for Ti (Titanium) in Grey Heron, *Ardea cinerea,* eggs from Barberspan*.* This may be because flamingos feed on organisms at lower trophic levels (Burger and Gochfeld 2001; Cosson et al. 1988; Rocha et al. 2021). However, the relatively low levels of most elements in flamingo eggs belie the fact that they are long lived (Hockey et al. 2005; Kear and Duplaix-Hall 1975), feed in highly saline waters rich in metal salts (Windisch et al. 2022), and therefore would be expected to have accumulated a bioaccumulative element such as mercury at higher concentrations than we found here. Their main food source, cyanobacteria, is known to accumulate Hg (Cain et al. 2008; Franco et al. 2018; Lefebvre and Budd 2007). The possible reasons for the unexpectedly low concentrations will be discussed below.

### Toxicity and Reproductive Implications

Toxicity reference values (TRVs) are expressions of toxicity associated with concentrations expected to be close to the toxicity threshold. They can be used for screening and baseline risk assessments (Beyer and Sample 2017). TRVs developed for metals in bird eggs by Meyer et al. (2014) are as follows: Cu 10–20 mg/kg dm; Hg 2 mg/kg dm; Se 7.7–15 mg/kg dm; Sr 66–73 mg/kg dm. A TRV of 176–507 mg/kg dm for Sr in eggshells was also set. Most concentrations of metals in this study were below the TRVs (Table 1). However, it should be noted that the TRVs are approximations and may not cover sensitive species.

The maximum Cu concentration in eggshells was 16 mg/kg, falling within the TRV range of 10–20 mg/kg. Copper is an essential nutrient for the growth and development of animals and is involved in various physiological and biochemical processes. However, concentrations as low as 3.5 mg/kg Cu induced oxidative stress in hens (Gou et al. 2021). The copper concentrations we found may, therefore, contribute towards the reproductive stress of *P. minor*.

Strontium concentrations in eggshells of *P. minor* (mean 840 mg/kg dm) exceeded the TRV set at 176–507 mg/kg dm. Strontium is passed from the blood and bones of the female to their eggs (Mora et al. 2011). Sr is a constituent of potential ecological concern along with Se, As, and Al (Meyer et al. 2014). When one element displays significant chemical similarity to an essential element, it can substitute it and potentially affect normal biological processes (Burger and Gochfeld 2001). High Ca requirements during egg production can cause an increase in Sr absorption (Kitowski et al. 2018). Ca, Sr, and Ba interact and can exert similar chemical and pharmacological effects (Mora et al. 2007). Concentrations of Sr may increase in embryos as they absorb the element from the eggshell (Mora et al. 2011). Bird nestling’s survival rate decreased with increased Sr concentration (Meyer et al. 2014). Mora (2003), Mora et al. 2007), and Mora et al. 2011) postulated that Sr could lead to embryo mortality and harmful effects such as egg breakage, reduced hatching success, and beak deformities by interfering with Ca metabolism and bone growth. Strontium may cause reproductive failure (Mora et al. 2011) and, in our case, may pose a reproductive risk to *P. minor*.

Windisch et al. (2022) found that conditions in a lake like Kamfers Dam (alkaline, saline, eutrophic, highly productive) were conducive to the methylation of Hg, leading to accelerated bioaccumulation and biomagnification. Hurter 2016 analysed metals in sediment from drainage depressions in South Africa. Considerable concentrations of Hg were recorded in some of these depressions where pans form. This is of concern as *P. minor* are known to frequent these pans (Hockey et al. 2005). In avian species, Hg has known toxic effects on developing embryos and the nervous system, especially in methylated form (MeHg) (Borghesi et al. 2011). Embryo health and survival decline at concentrations ranging from 0.2 mg/kg to 5.2 mg/kg dm across a broad range of bird taxa (Hargreaves et al. 2010). Rumbold et al. (2001) opined that egg content concentration is considered the best predictor of MeHg risk to avian reproduction. However, although Hg was quantified in egg contents in the present study, it was at concentrations below its TRV (the maximum in egg contents was 0.24 mg/kg dm).

There was also no significant correlation between Hg and Se concentrations in egg contents. Selenium detoxifies Hg by forming selenite (HgSe; molar ratio of 1:1), a non-toxic form of Hg (Koeman et al. 1973; Martoja and Berry 1980). We found no association between the two elemental concentrations in egg contents. However, the mean molar ratio we calculated at 8.2 (8.2 Se atoms for every Hg atom; minimum 3.1, maximum 20) suggests a highly protective effect afforded by Se. Selenium also affords protection against As and Cd (Becker 2000). Mercury, therefore, may only marginally contribute towards reproductive threats of the *P. minor* population that we measured in the eggs.

### Kamfers Dam Water Quality

Saline lakes accumulate various compounds (Christensen and Li 2014; McCulloch et al. 2008) and usually function as sinks for agricultural, industrial, and urban wastes (Valiente et al. 2022). The WWTP pumps treated and untreated sewage into Kamfers Dam (Hill 2016; Roos 2008b). This can result in poor water quality, elevated water levels, and hypereutrophication (toxic algal bloom events) (BirdLife SA 2018b; Hill et al. 2013). It can also increase the risk of disease outbreaks like avian botulism and affect the growth of *Arthrospira fusiformis*, the primary food source of *P. minor* (Hill et al. 2013; Ramollo 2018). A diagnosis of the avipox virus at Kamfers Dam was made in 2011 by Zimmermann et al. (2011). Toxic substances such as pesticides, toxic metals, and bacterial toxins, such as botulinum and cyanotoxins, have the potential to either kill or debilitate the flamingos. Their weakened state may make them more susceptible to bacterial or viral infections (Krienitz 2018). Pollution other than metals may therefore also contribute stress of the *P. minor* population breeding at Kamfers Dam.

### Mechanisms of Removal of Metals From the Bird’s Body

Phoenicopteridae are long-lived birds (Hockey et al. 2005; Kear and Duplaix-Hall, 1975). As they feed in areas where metal salts accumulate in saline lakes and seas, they would be expected to have higher egg burdens of metals through chronic exposure to water and food, which we did not find when compared with data of other freshwater bird eggs feeding at higher trophic levels (Table 2).

Metals are relatively easily absorbed as soluble salts (Eeva et al. 2020). Flamingos may ingest metals (dissolved, bound to particles, or accumulated in their food) directly as they filter feed (Beyer et al.^1999^; Nelson et al. 1998). This type of feeding behaviour exposes them directly to pollutants in sediments (Borghesi et al. 2017). Microbial reduction of sulphate to sulphide in anoxic sediments causes metals to become less soluble and less bioavailable (Manahan 2022). However, these salts may transfer directly across membranes upon contact (Manahan 2022), as is clearly the case for flamingos as they feed in saline waters (Fig. 2b; Sherry 2016).

Ingested metals are mobilised from tissues during nesting season and transported by blood or plasma proteins for excretion and/or incorporation into organs (Perrault et al. 2019). Avian species have developed mechanisms to get rid of salts, metabolic products, and pollutants through their feathers, guano, eggs and eggshells, salt glands, and oil glands (Burger 1994; Burger and Gochfeld 2004; Dauwe et al. 2005; Orłowski et al. 2017; Reshag et al. 2016; Schmidt-Nielsen 1997; Yamashita et al. 2021). Birds have uropygial (oil) glands, which are sebaceous glands that they use to preen themselves. The secretions from these glands have water-repellent properties (Salibian and Montalti 2009). It has been noted that aquatic birds tend to have larger glands than terrestrial species (Chiale et al. 2021). Among other physiological roles, the gland may protect birds exposed to pollutants, including metals, by secreting them onto their feathers during preening (Borghesi et al 2017; Salibian and Montalti 2009). Abdullah et al. (2015) found that Cd, Cr, and Pb in bird feathers may come from secretions of these glands. Some elements may accumulate in the keratin of feathers during their formation and development (Durkalec et al. 2022). Keratin, a sulphur-containing protein, has a high affinity for several metals (Squadrone et al. 2019).

Ebert et al. (2020) concluded that the life cycle dynamics of birds affect metal concentrations such as Hg over time and that larger, older, and healthier birds may bioaccumulate higher concentrations of these elements in their tissues. The fact that *P.minor* eggs have comparably low concentrations of metals (especially Hg; see also Table 2) needs consideration. Birds generally have a wider tolerance for changes in osmotic concentrations in their blood than mammals (McWilliams et al. 2016). Cosson et al. (1988) emphasised the importance of the liver and kidney as essential organs for the metabolism and excretion of trace elements.

In addition to kidneys, sweat, and digestive excretions, marine and desert birds also use salt glands to regulate salt and water balance, as do flamingos (Almansour 2007; Burger et al. 2000; Krienitz 2018; McWilliams et al. 2016). Salt glands eliminate salts from the bird through the bird’s nasal passages (Schmidt-Nielsen 1997). Burger et al. (2000) further suggest that metal cations actually concentrate in the salt gland and then secreted. Elements like Se may mimic other elements and, as a result, may also be taken up by the salt gland (Perrault et al. 2019). Burger and Gochfeld (2000) examined concentrations of metals in the salt glands of Laysan albatrosses (*Diomedea immutabilis*) and discovered that the salt gland had comparable concentrations of most metals to the kidney.

The salt gland and other means of elimination may, therefore, serve a significant excretory route of metals in flamingos (Burger et al. 2000; Burger and Gochfeld 2000; Perrault et al. 2019). Therefore, despite their long lifespans, these eliminatory mechanisms result in lower metal concentrations in their eggs when compared with other freshwater birds (Table 2). The accumulation and excretion of metals before oogenesis and embryogenesis may explain the lower-than-expected Hg concentrations in eggs. Although flamingo egg concentrations indicate some embryo exposure to metals, it is therefore unlikely that these concentrations reflect environmental contamination.

We should note the following: In eggs and tissues of *P. ruber* from Spain and *P. minor* from Kairu, non-polar organic pollutants were measured at appreciable concentrations (Guttiérez et al. 1997; Kairu 1994). This suggests that non-polar organic pollutants are less likely to be eliminated through salt glands compared with more polar salts. Non-polar pollutants may therefore be passed into eggs as they are less likely eliminated, potentially posing a higher threat compared with metals.

### Possible Re-exposure Routes

Within the first few days of hatching, flamingo chicks consume eggshells from their own and adjacent nests (Berry 1972). Berry (1972) analysed the stomach content of ten chicks, just mobile (3 – 4 days old), and they were found to be hard and packed with eggshell fragments. The average mass of eggshells per stomach was 0.8 g. Berry (1972) also reported that this behaviour is typical in this species, and ingestion may be because of high calcium demands for the growth of bones, especially the neck.

The remobilisation of metals from the eggshells via acidic action in the stomach, with a pH of 2.6 (Denbow 2000), occurs when chicks feed on their eggshells (since they use the Ca) and are likely to incorporate other metals from the eggshell matrix during this process. This form of post-hatching transfer (from mother to chick via shell) is a pathway of exposure and uptake of toxic metals not previously considered. Strontium, with high eggshell concentrations (Table 1), exhibits chemical mimicry with Ca (Mora et al. 2007). Chicks may be at risk of taking up Sr during ossification, as this element behaves similarly to Ca. Similarly, other metals (such as Cu, As, Al, and Cr – see section on toxicity) may also accumulate in or expose the chicks via eggshells.

Flamingo, hatchlings, nestlings, and fledgelings are fed with crop milk by their parents for up to six months (Ward et al. 2001). Exposure of young birds to parentally derived pollutants via crop milk (comparable to pollutants in mammalian milk) has not been studied. However, it is known that Greater Flamingo crop milk contains Ca, Na, Cu, Mn, and Mo (Ward et al. 2001) and most likely the other metals as well. The effectiveness of salt glands post-hatching is unknown. If under-developed, this may represent a period with a lack of defence against toxic metals compared with the preceding (seemingly) better-protected embryonic stage (Table 2). Crop milk should, therefore, be considered as an unrecognised mechanism of removal of pollutants from the parental body to the chick, in addition to those listed in the previous section, especially as feeding chicks with crop milk may last up to six months (Ward et al. 2001).

Metals from eggshell ingestion and parental crop milk consumption we identify here as previously unrecognised exposure routes to chicks.

### Potential Sources of Metals at Kamfers Dam

Flamingos may ingest metals as they filter for food (Beyer et al. 1999; Nelson et al. 1998). This type of feeding behaviour exposes them directly to pollutants in sediments (Borghesi et al. 2017; Gillingham et al. 2021). Metals can also adsorb to algal cells (Nelson et al. 1998). *P. minor* feed on *A. fusiformis*, which may accumulate certain trace elements (Muohi et al. 2018).

The untreated sewage water from the Homevale WWTP and stormwater runoff cause concern as they can introduce many contaminants, including metals, to the dam (Hill 2016; Roos 2008a, b, c, a,b,c). Roos (2008a) performed a water quality assessment on Kamfers Dam in 2008 February, July, and November, the year when the eggs were collected. In February 2008, the metal concentrations in Kamfers Dam sediments were much higher than in an unreferenced 2006 study. Some metals exceeded the guidelines for toxicants (particularly Al and Zn) in freshwater aquatic ecosystems. Al and Zn (see Table 1) were detected in eggshells, and the egg content of *P. minor.* Roos (2008b) reported that metal concentrations exhibited a notable decrease in July and were further reduced in November. These findings indicate the seasonal variability of metal concentrations in Kamfers Dam. This may be attributed to the fact that metal behaviour varies under natural conditions according to the chemical species of the metal, the presence of other metals and organic compounds, the volume of the receiving water, substratum type, dissolved oxygen, temperature, water hardness, pH, and salinity (Dallas and Day 2004; Nelson et al. 1998). Metals also alter their form from one speciation to another depending on their state (dissolved, suspended, and sorbed), which also influences their reactivity, bioavailability, and toxicity to aquatic organisms (Christensen and Li 2014; Nelson et al. 1998).

Due to the large variations in metal concentrations in 2008 at Kamfers Dam, we cannot connect the water and sediment concentrations with the *P. minor* egg concentrations. Flamingos could be exposed to sources beyond the dam.

## Conclusions and Recommendations

Kamfers Dam provides a valuable bird habitat characterised by extreme variations in water levels, physical and chemical conditions, pollution, and species composition (McColluch et al. 2008). It is the only known breeding site for Lesser Flamingos in South Africa (Colyn et al. 2024; Ramollo 2018), and it is threatened by various environmental stressors (Hill 2016) including metals.

This study was used to determine whether flamingo eggs can be used as effective biological indicators of metal pollution in Kamfers Dam. High concentrations of Sr and Cu were measured, which are known to cause adverse effects in birds. Other elemental concentrations were surprisingly low compared to those of other species from the same region. Due to their longevity and feeding habits, higher metallic concentrations, especially Hg, were expected in their eggs and eggshells. However, this was not the case. Because flamingos inhabit hostile environments, it seems they have evolved various metabolic pathways and physiological mechanisms to remove harmful substances taken up from their ambient environment prior to deposition in eggs, protecting the embryo. From a development point of view, post-hatching (hatchling, nestling, and fledgling) exposure and uptake via eggshell consumption and feeding with crop milk by the parents may represent a more sensitive developmental period than embryonic development.

To the best of our knowledge, this is the first report of metallic element concentrations in the egg content of any flamingo species. Based on our findings, flamingo eggs may not be good indicators of metal pollution in the environment and may not reflect the risk to the parental female. Lesser Flamingos also have a wide feeding and breeding range, further restricting their utility as indicators of environmental pollution. To infer the pollution of flamingos themselves, we suggest considering feathers, blood, organs, and salt and oil gland secretions for analyses. At least for flamingos, a more nuanced approach should therefore be adopted when considering exposure to and impacts of pollutants.
